# Probable Factors Associated with Response to Mesenchymal Stem Cell Therapy in Stroke Patients: A Post Hoc Analysis of the STARTING-2 Trial

**DOI:** 10.3390/jpm11111137

**Published:** 2021-11-02

**Authors:** Won Hyuk Chang, Jungsoo Lee, Jong-Won Chung, Yun-Hee Kim, Oh Young Bang, The STARTING-2 Collaborators

**Affiliations:** 1 Department of Physical and Rehabilitation Medicine, Center for Prevention and Rehabilitation, Heart Vascular Stroke Institute, Samsung Medical Center, Sungkyunkwan University School of Medicine, Seoul 06351, Korea; wh.chang@samsung.com (W.H.C.); jungsoo0319@gmail.com (J.L.); 2 Departments of Neurology, Samsung Medical Center, Sungkyunkwan University School of Medicine, Seoul 06351, Korea; jongwon.chung@samsung.com; 3 Translational and Stem Cell Research Laboratory on Stroke, Samsung Medical Center, Seoul 06351, Korea; 4 Department of Health Sciences and Technology, Department of Medical Device Management & Research, Department of Digital Health, SAIHST, Sungkyunkwan University, Seoul 06351, Korea

**Keywords:** mesenchymal stem cells, stroke, motor recovery, post hoc analysis, motor impairment

## Abstract

The aim of this study was to identify factors associated with improved motor function of the lower extremities in response to mesenchymal stem cell (MSC) therapy in patients with ischemic stroke. This study was a post hoc analysis of data from a prospective, open-label, randomized controlled trial of MSC therapy for patients with ischemic stroke patients associated with severe middle cerebral artery territory (STARTING-2 trial). Lower limb motor function was scored based on the lower limb of Fugl–Meyer assessment (FMA-LL) score before MSC therapy and at 3 months after stroke. All FMA-LL changes greater than or equal to six points were considered clinically significant. Univariate and multivariate binary logistic regression models were used to determine possible predictors of clinically significant lower limb motor response to MSC therapy. Twelve (33%) of the thirty-six patients receiving MSC therapy reached a minimal clinically important difference (MCID) of FMA-LL. The two independent factors with the greatest impact on response to MSC therapy for achieving an MCID in FMA-LL score were: (1) the time from stroke onset to MSC therapy, and (2) age (*p* < 0.05). In addition, obese stroke patients responded better to MSC therapy than stroke patients with normal weight. In conclusion, this post hoc analysis might suggest the need for recruiting stroke patients at younger and early after stroke onset in future clinical trials of MSC therapy for stroke.

## 1. Introduction

Many stroke survivors continue to suffer from significant motor impairments [1]. Ambulation is an essential part of disability in stroke patients. One-third to two-thirds of stroke survivors have reduced ambulatory capacity [2]. Impairment of lower extremity motor has been known as one of the major contributors to ambulatory disability after stroke [1,3]. Although most post-stroke care in the subacute phase depends on rehabilitation interventions, the improvement in lower extremity motor function, especially in patients with severe ischemic stroke, is still a challenge [4].

Stem cell therapy is an emerging paradigm in stroke treatment and is considered a potential regenerative strategy for patients with fixed neurologic deficits. Animal models of ischemic stroke have shown that stem cells transplanted into the brain can lead to functional improvement [5]. We have previously reported the results of the STARTING (STem cell Application Researches and Trials In NeuroloGy) trial, a randomized controlled trial of intravenous application of autologous mesenchymal stem cells (MSCs), culture-expanded with fetal bovine serum (FBS), in the subacute phase of stroke [6]. More recently, the STARTING-2 trial showed that MSC therapy improved lower extremity motor function in patients with subacute stroke with no significant adverse effects [7]. However, there was a relatively large standard deviation in the change of lower limb score of the Fugl–Meyer assessment (FMA-LL) that was measured as the lower extremity motor function in patients with stroke suggesting a highly variable response to MSC therapy. Multiple interacting factors are likely to affect motor recovery in stroke patients [8]; however, little is known about the factors that predict the efficacy of MSC therapy.

The aim of this study was to identify factors associated with improved motor function in response to MSC therapy in patients with ischemic stroke. The identification of these associated factors will enable the design of individual treatment plans and accurate stratification of patients for better outcomes after MSC therapy.

## 2. Materials and Methods

### 2.1. Study Design

This was a post hoc analysis of a prospective, open-label, randomized controlled trial with blinded outcome evaluation in patients with severe middle cerebral artery territory infarct investigated in the STARTING-2 trial [7]. The trial was registered at ClinicalTrials.gov (NCT01716481). The STARTING-2 trial protocol was approved by the Korean Food and Drug Administration (No. 12218) and the Institutional Review Board of the Samsung Medical Center (IRB-2011-10-047). Written informed consent was obtained from all patients and/or from their first-degree relatives.

Participants were randomly assigned in a 2:1 ratio to receive intravenous MSC injections (intervention group) or standard care alone (control group). After randomization, each participant received conventional rehabilitation during hospitalization. Participants in the intervention group underwent MSC treatment.

### 2.2. Participants

Of the 85 patients screened, 25 patients did not meet the inclusion or exclusion criteria and were excluded, and 60 patients were recruited. Of these, 15 patients were included in the control group and 39 in the MSC group were added to the final ITT analysis [7]. transcranial magnetic stimulation (TMS)-induced motor evoked potentials (MEPs) could not be evaluated in 3 of 39 patients in the MSC group due to craniotomy. Finally, this post hoc analysis was performed in 36 patients in the MSC group with ischemic stroke.

### 2.3. Post Hoc Analysis

The post hoc analysis evaluated the relationship between potential relating factors and change in FMA-LL from baseline to 3 months after MSC injection. Patients were stratified into good responders and poor responders, defined as patients achieving a minimal clinically important difference (MCID ≥ 6 points) of FMA-LL after MSC injection [9].

The potential associated factors were selected for this post hoc analysis because of their relating value in previous studies on motor recovery in stroke rehabilitation. For the baseline descriptive characteristics, age, sex, body mass index (BMI), and time since stroke onset were recorded at baseline in each patient [10,11,12,13]. Participants were classified into three groups based on their age: young (age <50 years), middle-age (50 ≤ age < 65 years), old age (≤65 years). In addition, based on obesity status, subjects were classified into four groups based on their baseline BMI: underweight (BMI < 18.5), normal (18.5 ≤ BMI < 23), overweight (23 ≤ BMI < 25), and obese (25 ≤ BMI) [14]. Based on time (time ≤ 30, 30 < time ≤ 60, 60 < time) since stroke onset, patients were divided into three groups. The National Institutes of Health Stroke Scales (NIHSS) NIH and FMA-LL score before MSC injection were used to represent baseline stroke severity and lower limb motor function, respectively. Stroke severity was classified into three groups based on baseline NIHSS: moderate (NIHSS < 9), moderate to severe (9 ≤ NIHSS < 16), and severe (16 ≤ NIHSS) [15]. In addition, to assess the integrity of the affected corticospinal tract TMS of the affected motor cortex was used to measure MEPs in the paretic first dorsal interosseous muscle at rest, as described previously [16]. Patients were classified into two groups according to the presence of MEPs on the affected first dorsal interosseus muscle (FDI): “MEPs response” included all patients who showed MEPs in the affected FDI, whereas “no MEPs response” included patients without any MEPs in the affected FDI.

### 2.4. Statistical Analysis

SPSS version 27.0 (SPSS, Chicago, IL, USA) was used for all statistical analyses. Good and poor responders were compared using an independent *t*-test and chi-square tests for normally distributed variables and Mann–Whitney tests for nonparametric data. Univariate logistic regression was conducted to identify possible factors associated with a good response to MSC therapy and any variables with univariate association with *p*-values < 0.20 were considered as potentially associated with MSC therapy and thus included in a multivariate model. Multivariate logistic regression models were then developed. A *p*-value < 0.05 was considered statistically significant.

## 3. Results

### 3.1. Comparison between Good and Poor Responders

**Figure 1:**
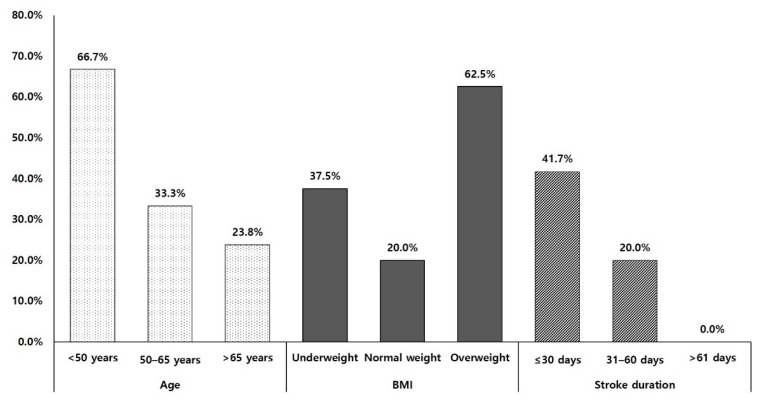
Rate of good responders. BMI: body mass index.

Twelve (33%) of the thirty-six patients receiving MSC therapy who reached the MCID of FMA-LL (≥6 points) were classified as good responders. By contrast, 24 (66.7%) patients were classified as poor responders. There was no significant difference in baseline demographics and clinical characteristics between good and poor responders in the MSC group (Table 1). The rate of good response showed a significant negative correlation with age and stroke duration (*p* < 0.05). In addition, obese stroke patients responded better to MSC therapy than stroke patients with normal weight (*p* < 0.05, Figure 1).

### 3.2. Associated Factors Analysis

Table 2 presents the results of univariate and multivariate analyses. In univariate analysis, age, stroke duration, and MEP response were factors associated with MCID of FMA-LL. Potential relating factors with a *p*-value < 0.2 were then used in multivariate analysis. Age and stroke duration were significant independent factors in the multivariate analysis predicting a good response with MCID of FMA-LL (*p* < 0.05, Nagelkerke’s R2 of 0.547, Table 2).

## 4. Discussion

A significant improvement in the affected lower extremity motor function without significant adverse effect was reported in the STARTING-2 trial of MSC therapy involving patients with middle cerebral artery infarction [7]. The two factors relating patient response to MSC therapy were age and stroke duration from onset to treatment. Therefore, these two factors could be considered in the design of MSC therapy for patients with ischemic stroke.

Several trials have been reported that intravenous autologous MSC therapy was safe and might improve motor recovery in subacute and chronic stroke patients [7,17,18]. There was a lack of reports on the factors affecting MSC therapy, although the treatment response might vary depending on the characteristics of the patients. It is necessary to identify the factors related to MSC therapy response for enhancing therapeutic efficacy. Stroke duration was analyzed as a significant factor independent of age determining the effects of MSC therapy in stroke patients in this post hoc analysis. Neuroplasticity has been suggested as a mechanism for improving motor function in subacute stroke patients [19]. Many pharmacotherapy trials have also demonstrated the effect of drugs on neuroplasticity in stroke patients [20]. Mechanistic targets of stem cells have been known as chemokines, trophic factors, and relevant microRNAs that increased markedly in the infarcted brain during the acute stroke phase and decreased with time [21,22]. These mechanistic targets are also important for neuroplasticity in stroke patients [22]. The mechanism of the motor function enhancing the effect of MSCs might be attributed to increased neuroplasticity. Therefore, stroke duration, which is a period of high neuroplasticity after stroke, was a significant independent factor affecting MSC therapy in this study [19].

In this post hoc analysis, age was one of the independent factors affecting MSC therapy among potential factors influencing motor recovery after stroke. This result might be attributed to age in predicting the prognosis of motor recovery in stroke patients [10]. However, age affects the MSC function of each participant. Aging has an impact on the function of stem cells because the general efficiency of most cellular and intercellular processes tends to decline with age [23]. In an animal study involving aging rodents, the proliferation of hypothalamic neural stem cells progressively declined in vivo and depleted eventually in aged mice [24]. In addition, the neurorestorative potential of MSCs may be limited in the elderly due to the limited number of bone marrow MSCs [25], and the relatively low number of neural stem/progenitor cells in the human brain [26]. To overcome these limitations, the STARTING-2 trial used the preconditioning MSCs using each patient’s serum to achieve rejuvenation of autologous MSCs. In spite of the preconditioning MSCs, the therapeutic effects were insufficient in aged stroke patients. Further studies with younger stem cells are needed to elucidate the mechanism associated with age underlying the effects of MSC therapy [22].

Stroke patients with overweight tended to be higher effective for achieving MCID of lower motor function than those with normal weight. Some previous studies have reported that obesity might be a positive influencing factor for functional outcomes after stroke [14,27]. This phenomenon, referred to as the obesity paradox, because obesity and overweight are well-known risk factors for the development of ischemic stroke [28]. The results of this study could also be considered as supporting results for the obesity paradox phenomenon in stroke patients. However, some studies have reported that there is insufficient evidence to support the obesity paradox controlling patient factors and comorbid conditions [29,30]. Therefore, further study will be needed to clarify the influence of obesity on the effects of MSCs therapy in stroke patients.

In this study, 33.3% of all patients who received MSCs showed MCID in their FMA-LL score. The relatively low rate may be attributed to the baseline severity of motor involvement in patients with stroke in the STARTING-2 trial. The most reliable factor predicting the prognosis of lower extremity motor function was baseline motor function in patients with subacute stroke [31]. Nevertheless, in this post hoc analysis, there was no relation between baseline FMA-LL and responders with MCID after MSC therapy for stroke, thereby indirectly demonstrating that MSCs were effective in improving spontaneous recovery.

This study has some limitations. First, we did not enroll patients with ischemic stroke showing mild motor involvement. Therefore, the study findings are not necessarily applicable to MSC therapy design to improve motor function in all patients with subacute stroke. Another limitation was that only patients who completed the follow-up were analyzed due to the nature of post hoc analysis. In addition, a relatively small number of patients was another limitation. Therefore, further studies with large numbers of patients diagnosed with stroke are needed.

## 5. Conclusions

In conclusion, younger age and a relatively short duration of stroke might be considered when individualizing MSC therapy to improve lower extremity motor function in patients with subacute stroke.

## Figures and Tables

**Table 1:** Baseline demographics and clinical characteristics of good responders and poor responders among stroke patients treated with mesenchymal stem cells.

Characteristics	Subgroup	Good Responders (*n* = 12)	Poor Responders (*n* = 24)	*p* Value
Age, mean (SEM)		60.7 (4.8)	66.6 (2.0)	0.48
	<50 years, *n* (%)	4 (33.3%)	2 (8.3%)	0.182
	50–65 years, *n* (%)	3 (25.0%)	6 (25.0%)	
	>65 years, *n* (%)	5 (41.7%)	16 (66.7%)	
Sex	Male, *n* (%)	6 (50.0%)	9 (37.5%)	0.499
	Female, *n* (%)	6 (50.0%)	15 (62.5%)	
BMI, mean (SEM)		24.5 (1.3)	22.2 (0.6)	0.115
	Underweight, *n* (%)	3 (25.0%)	5 (20.8%)	0.106
	Normal weight, *n* (%)	4 (33.3%)	16 (66.7%)	
	Overweight, *n* (%)	5 (41.7%)	3 (12.5%)	
NIHSS at baseline, mean (SEM)		11.1 (1.6)	12.3 (1.0)	0.511
	≤8, *n* (%)	4 (33.3%)	5 (20.8%)	0.813
	9–15, *n* (%)	5 (41.7%)	12 (50.0%)	
	≥16, *n* (%)	3 (25.0%)	7 (29.2%)	
Stroke duration, mean (SEM)		20.2 (3.4)	28.0 (4.8)	0.762
	≤30 days, *n* (%)	10 (83.3%)	14 (58.3%)	0.4
	31–60 days, *n* (%)	2 (16.7%)	8 (33.3%)	
	>61 days, *n* (%)	0 (0.0%)	2 (8.3%)	
FMA-LL at baseline, mean (SEM)		7.3 (2.0)	9.0 (1.4)	0.156
MEPs response	Yes	1 (8.3%)	7 (29.2%)	0.224
	No	11 (91.7%)	17 (70.8%)	

BMI: body mass index; NIHSS: National Institutes of Health Stroke Scale; FMA-LL: lower limb score of Fugl–Meyer assessment; MEPs: motor evoked potentials.

**Table 2:** Univariate logistic regression analysis of the association between baseline clinical features with responders in lower limb score of Fugl–Meyer assessment of patients treated with stem cells.

Potential Relating Factors	Good Responders			
	Univariate Analysis		Multivariate Analysis	
	Exp(β) (95% CI)	*p* Value	Exp(β) (95% CI)	*p* Value
Age group	0.425 (0.167–1.083)	0.073	0.264 (0.070–0.994)	0.049 *
Sex	1.447 (0.410–6.767)	0.475	NS	NS
BMI group	2.400 (0.396–14.556)	0.341	NS	NS
NIHSS group at baseline	0.727 (0.277–1.910)	0.518	NS	NS
Stroke duration group	0.294 (0.060–1.440)	0.131	0.067 (0.006–0.786)	0.031 *
FMA-LL at baseline	0.959 (0.856–1.076)	0.478	NS	NS
MEPs response	0.221 (0.024–2.050)	0.184	0.056 (0.002–1.643)	0.095

BMI: body mass index; NIHSS: National Institutes of Health Stroke Scale; FMA-LL: lower limb score of Fugl–Meyer assessment; MEPs: motor evoked potentials. * *p* < 0.05.

## Data Availability

The data that support the findings of this study are available from the corresponding author upon reasonable request.

## Appendix A

STARTING-2 Investigators: Oh Young Bang, MD, PhD; Jong-Won Chung, MD, PhD; Suk Jae Kim, MD, MSc; Soo-Kyoung Kim, MD, PhD; Jin Soo Lee, MD, PhD; Sung-Il Sohn, MD, PhD; Yun-Hee Kim, MD, PhD; Won Hyuk Chang, MD, PhD; Jungsoo Lee, PhD; Yeon Hee Cho, MS; Ji Hee Sung, MS; Eun Hee Kim, PhD; Jeong Pyo Son, PhD; Dong Hee Kim, PhD; Eun-Hyeok Choi, MD; Sookyung Ryoo, MD; Yoon Mi Kang, MS; Yong Man Kim, PhD; Hyun Soo Kim, MD, PhD; Jun Ho Jang, MD, PhD.
